# mRNAsi-related metabolic risk score model identifies poor prognosis, immunoevasive contexture, and low chemotherapy response in colorectal cancer patients through machine learning

**DOI:** 10.3389/fimmu.2022.950782

**Published:** 2022-08-23

**Authors:** Meilin Weng, Ting Li, Jing Zhao, Miaomiao Guo, Wenling Zhao, Wenchao Gu, Caihong Sun, Ying Yue, Ziwen Zhong, Ke Nan, Qingwu Liao, Minli Sun, Di Zhou, Changhong Miao

**Affiliations:** ^1^ Department of Anesthesiology, Zhongshan hospital, Fudan University, Shanghai, China; ^2^ Shanghai Key Laboratory of Perioperative Stress and Protection, Zhongshan hospital, Fudan University, Shanghai, China; ^3^ Department of Pathology, Obstetrics and Gynecology Hospital, Fudan University, Shanghai, China; ^4^ Department of Diagnostic and Interventional Radiology, University of Tsukuba, Ibaraki, Japan; ^5^ Department of Diagnostic Radiology and Nuclear Medicine, Gunma University Graduate School of Medicine, Maebashi, Japan

**Keywords:** colorectal cancer, mRNAsi, stemness, risk score model, immunotherapy, metabolism, immune evasion, Machine learning

## Abstract

Colorectal cancer (CRC) is one of the most fatal cancers of the digestive system. Although cancer stem cells and metabolic reprogramming have an important effect on tumor progression and drug resistance, their combined effect on CRC prognosis remains unclear. Therefore, we generated a 21-gene mRNA stemness index-related metabolic risk score model, which was examined in The Cancer Genome Atlas and Gene Expression Omnibus databases (1323 patients) and validated using the Zhongshan Hospital cohort (200 patients). The high-risk group showed more immune infiltrations; higher levels of immunosuppressive checkpoints, such as CD274, tumor mutation burden, and resistance to chemotherapeutics; potentially better response to immune therapy; worse prognosis; and advanced stage of tumor node metastasis than the low-risk group. The combination of risk score and clinical characteristics was effective in predicting overall survival. Zhongshan cohort validated that high-risk score group correlated with malignant progression, worse prognosis, inferior adjuvant chemotherapy responsiveness of CRC, and shaped an immunoevasive contexture. This tool may provide a more accurate risk stratification in CRC and screening of patients with CRC responsive to immunotherapy.

## Introduction

Colorectal cancer (CRC) is one of the deadliest cancers of the digestive system (1, 2). Although there is an increasing number of potential therapeutic approaches for CRC, such as surgery, chemotherapy, radiotherapy, and molecular targeted therapy, the clinical prognosis remains unsatisfactory, especially for patients with distant metastasis of CRC (3, 4). Therefore, accurate medical treatment is essential for the eradication of malignancy. At the same time, due to the high molecular heterogeneity of CRC, most existing biomarkers lack strong predictive accuracy (5). Hence, it has become an urgent problem to find a powerful index to predict and evaluate the clinical prognosis and therapeutic effect to achieve accurate clinical intervention.

Cancer stem cells (CSCs) play a crucial part in the progression, recurrence, and drug resistance of solid malignant tumors (6). Furthermore, CSCs promoted immunosuppression, immune escape, tumor metastasis, and therapeutic resistance by interacting with immune cells (7). For example, in a co-transplantation environment, CSCs can promote the polarization of CD14^+^ peripheral monocytes into immunosuppressive M2 macrophages and the generation of tumorigenic myeloid cells, followed by the acceleration of tumor growth in immunocompromised mice (8). CSCs also drive the recruitment and polarization of TH17 cells and Treg cells by secreting CCL1, CCL2, CCL5, and TGF-β, resulting in an immunosuppressive environment (7). In recent years, the mRNA expression-based stemness index (mRNAsi) developed by a machine learning algorithm has been used to quantify the stemness characteristics of tumors (9) such as esophageal cancer (10), gastric cancer (11), hepatocellular carcinoma (12), and glioma (13). However, the risk score model for stemness features associated with immunological properties in CRC remains uninvestigated.

Metabolic reprogramming, a hallmark of cancer, is another important factor leading to antitumor immunity and immune escape. For example, excessive glycolysis in tumor cells produces a large amount of lactate, which leads to acidification of the microenvironment and, consequently, inhibits the proliferation and function of cytotoxic T cells. Other studies also showed that inhibition of mTOR or the glycolysis pathway regulated T-cell differentiation into naïve and memory phenotypes (14). Furthermore, when CAR-T cells were expanded *in vitro*, inhibition of AKT improved their metabolism and promoted their differentiation to the memory phenotype, thus improving the progression of acute lymphoblastic leukemia (15). Therefore, further elucidation of the effects of tumor stemness and metabolic characteristics on the immune microenvironment may provide significant clinical benefits.

In this study, we generated a new risk prediction model using mRNAsi and metabolism-related genes using CRC expression data retrieved from The Cancer Genome Atlas (TCGA) and Gene Expression Omnibus (GEO) databases (n = 1323). We also determined the association between the risk score and several functional and clinical features of patients with CRC. Clinical prognosis, tumor microenvironment and immunophenotype, response to chemotherapy and immunotherapy, and genomic variation between two risk score groups were evaluated comprehensively. We then validated the mRNAsi-related metabolic risk score model using the Zhongshan Hospital cohort (n = 200). Our data aimed to provide new insights into the screening of patients more likely to benefit from immunotherapy, and to improve individualized treatments for CRC patients.

## Methods

### Data collection and processing

The expression profile data of colon adenocarcinoma (COAD) and rectal adenocarcinoma (READ) of 591 patients, and their clinicopathological annotation were retrieved from the TCGA GDC website (https://portal.gdc.cancer.gov/). TPM values were converted from FPKM. Furthermore, tumor mutation burden (TMB) was obtained by analyzing the copy number variation (CNV) and somatic mutation data using the maftools package of R.

In addition, CRC gene expression data of GSE17536 (16–18) and GSE39582 (19) and the clinicopathological features of the patients were also downloaded from the GEO database. GSE17536 included 177 CRC tissue samples, and GSE39582 included 555 CRC tissue samples. Subsequently, the TCGA and GEO data were merged (n = 1323), and the limma (20) R package and sva (21) R package were used to combine and eliminate any batch effect.

### Analysis of mRNAsi and differentially expressed genes

Based on the relative expression data provided by Zheng et al. (22), the mRNAsi of each sample was determined conforming to the gene expression matrix by the single-sample gene-set enrichment analysis (ssGSEA) method using the R-package GSVA. According to the mRNAsi of each sample obtained, combined with the survival status of the patients, the best cut-off value of mRNAsi was set, and the patients with CRC were distributed into the high-mRNAsi group and low-mRNAsi group.

DEGs between the high-mRNAsi groups and low-mRNAsi groups in patients with CRC were analyzed using the “limma” R package. DEGs were defined as genes with Log2 (fold change) > 1.0 and *P <* 0.05. Metabolism-related gene sets were copied from the Molecular Signature Database (MSigDB) V7.0 (22). Finally, the overlap between DEGs and metabolism-related genes resulted in the identification of metabolism-related DEGs.

### Weighted gene co-expression network analysis

WGCNA was achieved using the WGCNA package in R, which aims to determine the correlation between genes by building important modules. First, a scale-free gene co-expression network was constructed according to the weight of the correlation coefficient, and a hierarchical clustering tree was established depending on the adjacency matrix of the network. The module significance (MS) was then calculated to judge the correlation between the mRNAsi value and different modules. The genes in each module were recorded and defined as module characteristic genes. Modules with maximum and minimum MS values were regarded as positive and negative modules, respectively. After selecting the modules of interest according to the MS values, all gene expressions in the modules were identified as genes highly correlated with mRNAsi.

### Construction of mRNAsi-related metabolic risk score model

By integrating the results of metabolism-related DEG analysis and WGCNA, mRNAsi-related metabolic genes were finally obtained. Significantly differentially expressed mRNAsi-related metabolic genes were included in the model, dimensionality reduction analysis was performed using the minimum absolute contraction and selection operator (least absolute shrinkage and selection operator, LASSO) algorithm, and the characteristic genes related to prognosis were obtained. Using the normalized gene expression value weighted by the penalty coefficient obtained by LASSO Cox analysis, a risk score formula was established, and patients were divided into high-risk group and low-risk group according to the median risk score.


$$Risk\;score\;=\;\sum_{i}Coefficient\;\left(hub\;gene_{i}\right)\times mRNA\;Expression\;\left(hub\;gene_{i}\right)$$

### Functional and pathway enrichment analyses

Gene Ontology (GO) analysis is a widely used method for functional enrichment studies and generates data related to biological processes (BP), molecular functions (MF), and cellular components (CC). Kyoto Encyclopedia of Genes and Genomes (KEGG) is a database for systematic analysis of gene function, linking genome information with more orderly biological function information. The clusterProfiler package of R (23) was used for GO analysis and KEGG pathway enrichment in the mRNAsi-related metabolic risk score model. FDR < 0.05 was regarded as significant.

To investigate differences in BP between different groups, we employed gene-set enrichment analysis (GSEA) (22). The “h.all.v7.2.symbols.gmt” gene set was copied from the MSigDB for the GSEA. *P <* 0.05 was considered significant.

### Molecular network analysis

The STRING database (https://cn.string-db.org) (24) was used to construct a protein-protein interaction (PPI) network. Genes with scores greater than 0.4 were chosen to build a network model, which was visualized using Cytoscape (v3.7.2) (25). Then, eight hub genes were selected using the CytoHubba plug-in (26) in the Cytoscape software. Furthermore, we use the GOSemSim package in R (27) to judge the GO semantic similarity of the eight genes (28).

Information regarding miRNA-mRNA interactions from the miRTarBase database was downloaded before analyzing the basic statistics. Based on the core mRNA obtained by PPI analysis, the miRTarBase database was used to predict the miRNAs that may be regulated and to further predict the related lncRNAs. Cytoscape software was used to visually display the results of ceRNA analysis.

### Analysis of tumor immune infiltrating cells

An ssGSEA algorithm was deployed to measure the relative number of tumor-infiltrating immune cells in patients with CRC (29). The enrichment score calculated by ssGSEA using the GSVA R package (30) indicates the in level of each immune cell type in each sample. In addition, depending on the gene expression profile, the ESTIMATE R package (31) was used to quantify the level of immune infiltration of tumor samples, and the immune score of each tumor sample was obtained. The differences in the immune infiltration characteristics of CRC patients between the high-risk score group and low-risk score groups were evaluated.

### Analysis of drug sensitivity and immunotherapy response

The Genomics of Drug Sensitivity in Cancer (GDSC) (https://www.cancerrxgene.org/) is an open database for molecular therapy and mutation exploration in cancer. The pRRophetic package of R (32) was used to download the cell line gene mutation data and the IC50 values of different anticancer drugs from GDSC (33) and to analyze the correlation between patients with high and low-risk scores and different anticancer drug sensitivities.

In addition, we used online tumor immune dysfunction and exclusion (TIDE) scores (34) to examine immunotherapy sensitivity and compare the scores of tumor immunotherapy markers, such as CD8 and CD274, between the high-risk score groups and the low-risk scoring groups. The response of immune-checkpoint blockade was predicted.

### CNV analysis

To analyze the changes in copy number in different risk score groups of patients with TCGA-CRC, we used the TCGAbiolinks package of R to obtain the masked copy number segment data of the patients. The downloaded CNV fragments were analyzed using GISTIC 2.0, with default settings in GenePattern5. Finally, the analysis results of GISTIC 2.0 were visualized through the maftools package of R.

### Establishment of a prognostic model

Univariate and multivariate Cox analyses were used to predict the overall survival (OS) of patients with CRC. The clinicopathological features were then incorporated into the risk score model to construct a clinical predictive nomogram. To quantify the differential performance of the nomogram, Harrell’s consistency index (C-index) was estimated. A calibration curve was produced and the capability of the nomogram was evaluated.

### Patients and CRC tissue samples

The Zhongshan Hospital cohort included 200 patients who underwent CRC surgery between January 2008 and December 2014. The patients’ baseline characteristics included sex, age, adjuvant chemotherapy, tumor location, tumor histology, tumor differentiation, nerve invasion, surgical margin positivity, and stage of tumor node metastasis (TNM). Tumor staging was performed according to the 7th edition of the American Joint Commission on Cancer (AJCC) TNM Classification (35). Conforming to the National Comprehensive Cancer Network guidelines and patient wishes, patients with stage III-IV TNM were treated with ACT after surgery. OS was described as the time from the date of diagnosis to death or last follow-up. Disease-free survival (DFS) was described as the time from the date of diagnosis to relapse or last follow-up. The follow-up period ended on December 31, 2020. Clinical data validation was approved by the ethics committee of the Zhongshan Hospital (B2022-068R2).

### RNA separation and quantitative reverse transcription PCR

The mRNA expression of 21 mRNAsi-related metabolic genes was measured by qRT-PCR in Zhongshan cohort. Total RNA was obtained using the TRIzol reagent (Invitrogen, Waltham, MA, USA). cDNA was obtained by reverse transcription using the PrimeScript RT kit (Takara). The expression of candidate genes and the housekeeping gene GAPDH was evaluated by quantitative reverse transcription PCR using the ABI 7900HT real-time PCR system (Applied Biosystems, Carlsbad, CA, USA). Relative transcription levels were calculated using the ΔΔCt method (36). The primer sequences used are listed in **Supplementary Table 7**.

### Immunohistochemical staining

We randomly selected 20 cases from 200 Zhongshan patients for IHC, including 10 cases in high risk group and 10 cases in low risk group. Paraffin-embedded tissues were stained with antibodies. The staining score was decided by two experienced pathologists at the Zhongshan Hospital. Six high-power fields (HPFs, ×200 magnification) were randomly counted by two independent pathologists (each with three fields), and the densities of CD8^+^T cells, Foxp3^+^Tregs, CD19^+^B cells, CD11c dendritic cells, immunosuppressive checkpoints (PD-1, PD-L1) and effector molecules (GZMB, PRF1) were recorded. Immunohistochemistry antibodies are listed in **Supplementary Table 8**.

### Statistical analysis

All data processing and analyses were accomplished using the R software (version 3.6.2) and SPSS (version 25; IBM, Armonk, USA). For the comparison of two groups of continuous variables, the statistical significance of normally distributed variables was calculated using an independent *t*-test, and the difference between non-normally distributed variables was measured using the Mann–Whitney U test. Chi-square test or Fisher’s exact test was used to analyze the significant differences between the two groups of classified variables. The survival package in R was conducted for survival analysis. The receiver-operating characteristic (ROC) curve was drawn by the pROC package of R (37) and the area under the curve (AUC) was calculated to evaluate the performance of the risk score model. Univariate and multivariate Cox analyses were used to determine independent prognostic factors. All statistical P values were bilateral, and *P < 0.05, **P < 0.01, ***P < 0.001 were regarded as statistically significant.

## Results

### Relationship between colorectal cancer stemness characteristics and clinical features

**Figure 1:**
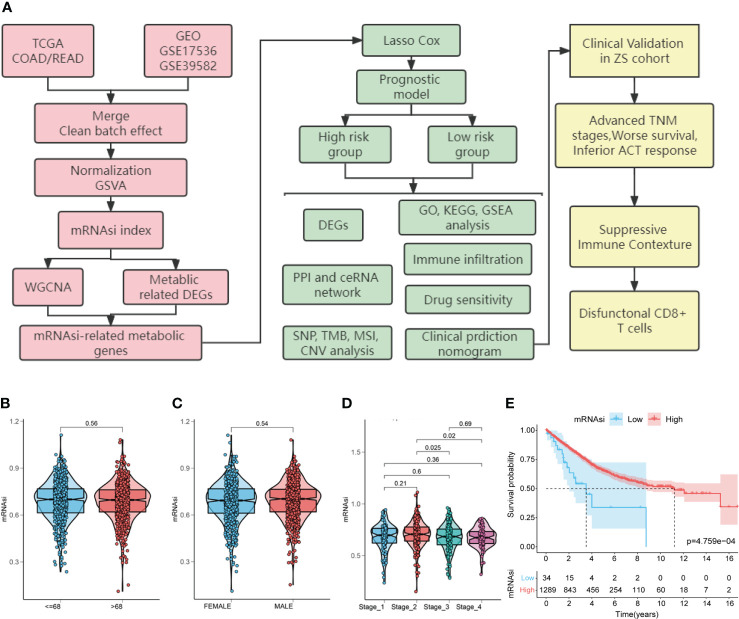
Study flow chart and the relationship between colorectal cancer (CRC) stemness characteristics and clinical features. **(A)**. Flow chart for construction and validation of mRNAsi-related metabolic risk score model in CRC. TCGA, The Cancer Genome Atlas; GEO, Gene Expression Omnibus databases; mRNAsi, mRNA expression-based stemness index; DEGs, differentially expressed genes; WGCNA, weighted gene co-expression network analysis; GO: gene ontology; KEGG, Kyoto Encyclopedia of Genes and Genomes; GSEA, gene-set enrichment analysis; PPI, protein-protein interaction; ceRNA, competing endogenous RNAs; SNP, single nucleotide polymorphism; TMB, tumor mutation burden; MSI, microsatellite instability; CNV, copy number variation; TNM, tumor node metastasis; ACT, adjuvant chemotherapy. **(B–E)**. Relationship between CRC stemness characteristics and clinical features. Analysis of the correlation of mRNAsi with age **(B)**, gender **(C)**, TNM stage **(D)** and overall survival **(E)** in patients with CRC.

A flowchart of this study is shown in **Figure 1A**. To explore the role of mRNAsi on the progression of CRC, including COAD and READ, the gene expression matrices of GSE17536 and GSE39582 datasets and TCGA database were downloaded (**Supplementary Figures 1A, B**). The data from the two databases were then merged (n = 1323) and cleaned from any batch effect (**Supplementary Figures 1C, D**).

First, to explore the correlation between mRNAsi and clinical characteristics, we determined CRC mRNAsi using the ssGSEA algorithm. Then, according to the optimal mRNAsi cut-off value, the patients with CRC were separated into high-mRNAsi and low-mRNAsi groups. The relationship between CRC stemness characteristics and clinical characteristics is shown in **Figures 1B-E**. No significant correlation between mRNAsi and age (*P =* 0.56) or gender (*P =* 0.54) was observed (**Figures 1B, C**). However, higher mRNAsi were associated with staging of TNM (stage 2 vs. stage 3, *P =* 0.025; stage 2 vs. stage 4, *P =* 0.02; **Figure 1D**). Furthermore, patients with high mRNAsi showed a significant increase in OS compared to those with low mRNAsi (log-rank *P <* 0.001, **Figure 1E**).

### Identification of mRNAsi-related metabolic genes in patients with CRC

**Figure 2:**
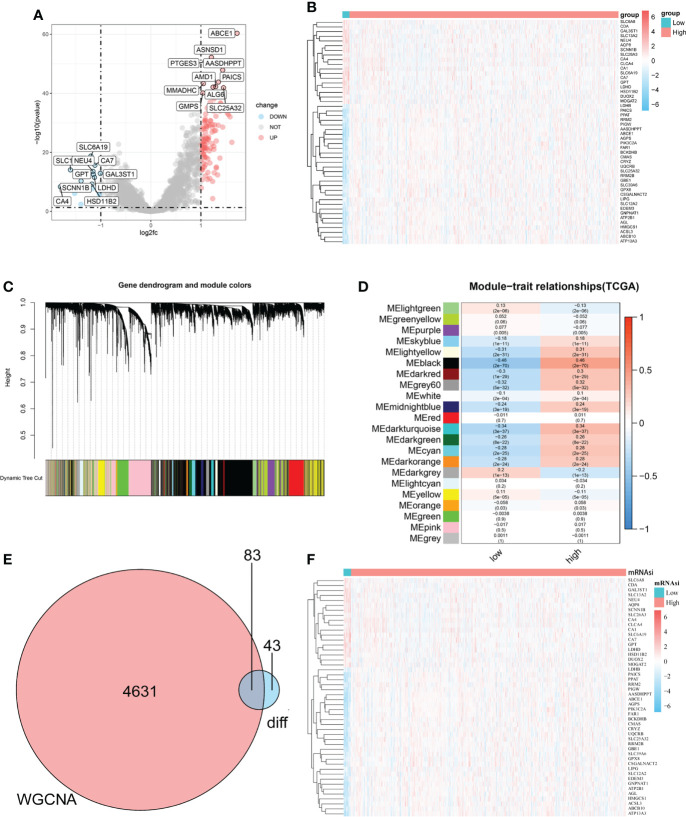
Identification of mRNAsi-related metabolic genes in patients with CRC. **(A–B)**. Volcano plot and heatmap showing the expression of metabolism-related DEGs in patients with CRC. **(C)**. Genes with similar expression patterns were merged in the same module to create a hierarchical cluster tree. **(D)**. Correlations and significant differences between different gene modules and mRNAsi and P values are displayed in the module. **(E)**. All genes in the black module, which were more closely related to mRNAsi, were overlapped with metabolism-related genes and 83 candidate genes were obtained, which were defined as mRNAsi-related metabolic genes. **(F)**. The heatmap shows the expression of 83 significantly differentially expressed mRNAsi-related metabolic genes in the CRC and normal tissues.

To determine the role of the mRNAsi in metabolic processes in CRC, DEGs between the high-mRNAsi and low-mRNAsi groups were identified and intersected with a metabolic gene set (2752 genes). One hundred and twenty-six genes were obtained and labeled as metabolism-related DEGs, of which 108 genes were significantly upregulated, and 18 genes were significantly downregulated (**Figures 2A, B** and **Supplementary Figure 3A**). WGCNA was used to identify modules closely related to mRNAsi-related genes. A total of 22 co-expression modules were identified, with the black module showing the strongest correlation with mRNAsi in CRC (**Figures 2C, D**). All genes in the black module were intersected with the metabolism-related DEGs, and 83 mRNAsi-related metabolic genes were obtained for further analysis, as shown in the Venn diagram (**Figures 2E, F** and **Supplementary Figure 3B**).

### Construction of mRNAsi-related metabolic risk score model

**Figure 3:**
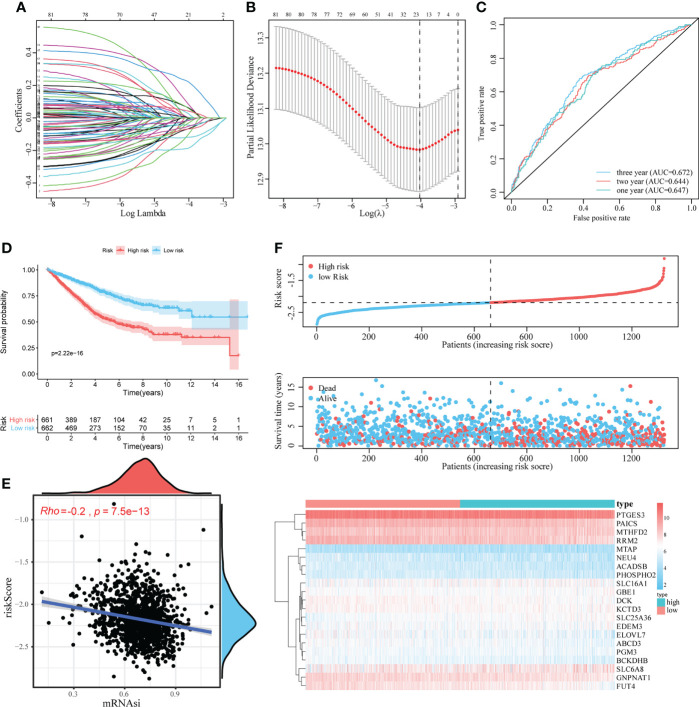
Construction and evaluation of the mRNAsi-related metabolic risk score model. **(A, B)**. LASSO Cox analysis identified 21 genes most associated with OS in the TCGA dataset. **(C)**. Time-dependent ROC curve analysis of risk score. **(D)**. The effect of the risk score assessed by Kaplan–Meier curve on the overall survival rate of patients with CRC. **(E)**. Spearman rank correlation analysis was used to analyze the relationship between mRNAsi and risk score. **(F)**. The risk score distribution, survival status, and heatmap of characteristic gene expression in patients with CRC.

To quantitatively evaluate the predictive value of identified mRNAsi-related metabolic genes in the clinical prognosis of CRC, we constructed a risk score model based on these genes. First, the expression characteristics of the 83 mRNAsi-related metabolic genes were included in the LASSO Cox analysis and 21 genes with the optimal predictive value were selected (**Figures 3A, B**). Simultaneously, a risk score formula was established based on the normalized expression of important characteristic genes weighted by the penalty coefficient calculated by LASSO Cox analysis, and a risk score for each sample was calculated. An example of the formula used to calculate the risk score is given below.


$$\begin{array}{l} \text{Risk score}=\left(-0.1053\right)\times \text{PTGES}3+\left(-0.1874\right)\times \text{PAICS}+\left(-0.0133\right)\times \text{GNPNAT}1+\\ \left(0.02893\right)\times \text{PGM}3+\left(0.08862\right)\times \text{MTHFD}2+\left(-0.0043\right)\times \text{DCK}+\left(0.06428\right)\times \text{MTAP}+\\ \left(0.22468\right)\times \text{SLC}25A36+\left(0.00308\right)\times \text{GBE}1+\left(-0.0679\right)\times \text{RRM}2+\left(0.0029\right)\times \text{KCTD}3\\ +\left(-0.0436\right)\times \text{ACADSB}+\left(-0.0339\right)\times \text{ABCD}3+\left(-0.0137\right)\times \text{BCKDHB}+\left(-0.0525\right)\times \\ \text{PHOSPHO}2+\left(-0.1185\right)\times \text{FUT}4+\left(0.00089\right)\times \text{EDEM}3+\left(-0.0684\right)\times \text{NEU}4+\left(0.6165\right)\\ \times \text{SLC}16A1+\left(-0.0162\right)\times \text{ELOVL}7+\left(0.04312\right)\times \text{SLC}6\text{A}8 \end{array}$$

Then we performed the time-dependent ROC curve analysis and found that the model had appropriate accuracy in predicting OS in patients with CRC, and the AUC of 1-year, 2-year and 3-year OS was 0.647, 0.644, and 0.672, respectively (**Figure 3C**). Kaplan–Meier analysis showed a reduction in OS in patients with high-risk scores (log-rank *P <* 0.001; **Figure 3D**). In addition, there was a significant negative correlation between mRNAsi and risk scores (Rho = -0.2, *P <* 0.001, **Figure 3E**). The distribution of the risk score, survival status, and expression pattern of characteristic genes is shown in **Figure 3F**.

### GSEA, GO, KEGG analyses of DEGs between high-risk and low-risk patients in mRNAsi-related metabolic risk score model

**Figure 4:**
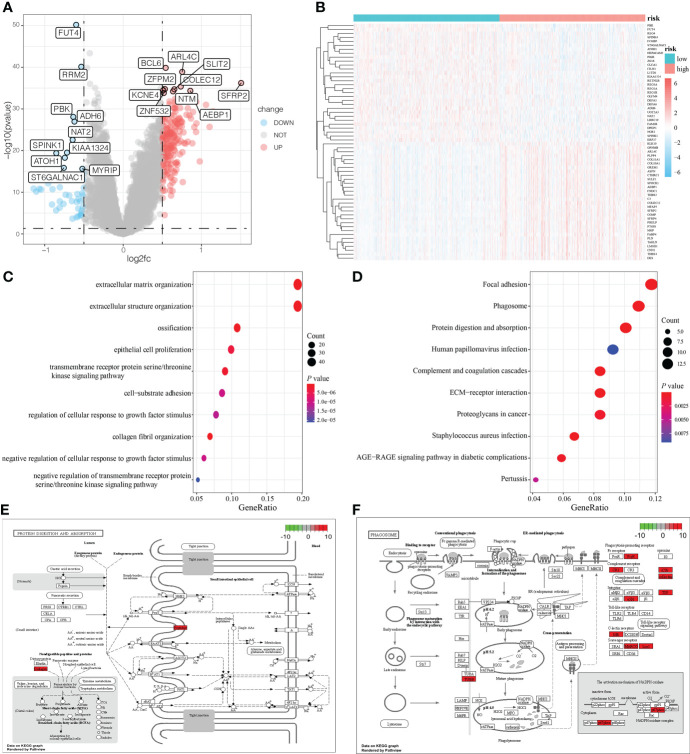
DEG analysis and functional enrichment analysis based on the mRNAsi-related metabolic risk score model. **(A, B)**. Volcano plot and heatmap showing the expression pattern of DEGs in two groups of patients with CRC. **(C, D)**. Biological processes (BP) and KEGG pathway analysis of DEGs in two groups of patients with CRC. **(E, F)**. The two pathways are closely related to the mRNAsi-related metabolic risk score model: protein digestion and absorption, and the phagosome pathway.

To analyze the impact of mRNAsi-related metabolic risk score models on the occurrence and development of CRC, we used the median LASSO Cox risk score of CRC cases from TCGA dataset and divided the CRC cases into high-risk and low- risk score groups. There were 242 DEGs between high-risk and low-risk patients (Log2 (fold change) > 1.0 and *P <* 0.05), of which 195 were significantly upregulated, and 47 were significantly downregulated (**Figures 4A, B**). The correlation between the risk score with the clinical characteristics of CRC patients in the TCGA and GEO database is shown in **Supplementary Table 1**. The functional annotation of the GO showed the DEGs were closely related to several BP, including the organization of the extracellular matrix, the organization of the extracellular structure and ossification, as well as several MF such as extracellular matrix structural constituent, glycosaminoglycan binding, and extracellular matrix structural constituent conferring tensile strength (**Figure 4C**, **Supplementary Table 2**). KEGG analysis indicated that DEGs were particularly involved in focal adhesion, phagosome, protein digestion and absorption, complement and coagulation cascades, and ECM-receptor interaction pathways (**Figure 4D** and **Supplementary Table 3**). Two pathways, protein digestion and absorption (*P =* 2.82E^-08^) and phagosome pathways (*P =* 2.96E^-07^), which were highly related to the mRNAsi-related metabolic risk score model, are shown in **Figures 4E, F**.

Furthermore, GSEA showed that ascorbate and aldarate metabolism (NES = -1.89, *P =* 0.002), citrate cycle (TCA cycle) (NES = -2.28, *P =* 0.002), glyoxylate and dicarboxylate metabolism pathways (NES = -2.03, *P =* 0.002), propanoate metabolism (NES = -2.07, *P =* 0.002), arginine and proline metabolism (NES = -1.94, *P =* 0.002), pyruvate metabolism (NES = -2.18, *P =* 0.002), hallmark oxidative phosphorylation (NES = -2.86, *P <* 0.001), and hallmark fatty acid metabolism (NES = -1.93, *P <* 0.001), were abundant in low-risk patients, whereas hallmark hypoxia was significantly enriched in high-risk patients (NES = 2.04, *P <* 0.001), (**Supplementary Figures 2A–I** and **Supplementary Table 4**).

### Construction of PPI network and related regulation network

**Figure 5:**
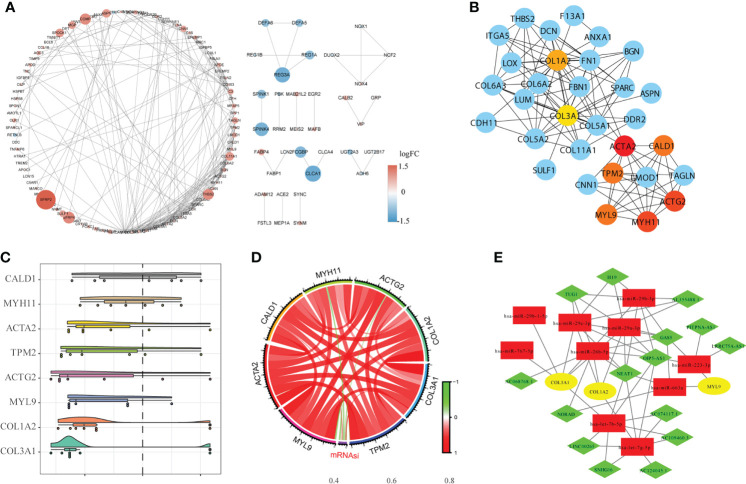
Construction of the protein-protein interaction network (PPI) and the ceRNA network. **(A)**. The results of PPI analysis were introduced into Cytoscape software for analysis, in which red represents upregulated genes, blue represents downregulated genes, and color depth and node size were positively correlated with log fold change (FC). **(B)**. The CytoHubba algorithm was used to identify and extract the top eight genes from the PPI network as the hub genes. **(C)**. GO semantic similarity analysis of importance of the eight hub genes. **(D)**. Circle diagram of the correlation between the hub gene and mRNAsi. **(E)**. Construction of the ceRNA interaction network based on hub genes.

We used the STRING database to establish the PPI network between DEGs, and imported the interaction between genes into Cytoscape software to obtain **Figure 5A**, in which the upregulated genes were represented in red and the downregulated genes were represented in blue.

The hub genes were analyzed using Cytoscape software (**Figure 5B**). GO semantic similarity analysis showed that the *CALD1* gene played an important role in the hub genes (**Figure 5C**). Subsequently, we conducted correlation analysis between hub genes and mRNAsi, and found a significant co-expression pattern between hub genes, whereas the relationship of each hub gene and mRNAsi was not consistent (**Figure 5D**). Finally, based on information about miRNA-mRNA interaction downloaded from the miRTarBase database; the hub genes obtained *via* the PPI network were used to construct the ceRNA network of miRNA-mRNA–lncRNA interaction (**Figure 5E**).

### Immune contexture difference between high-risk and low-risk patients

**Figure 6:**
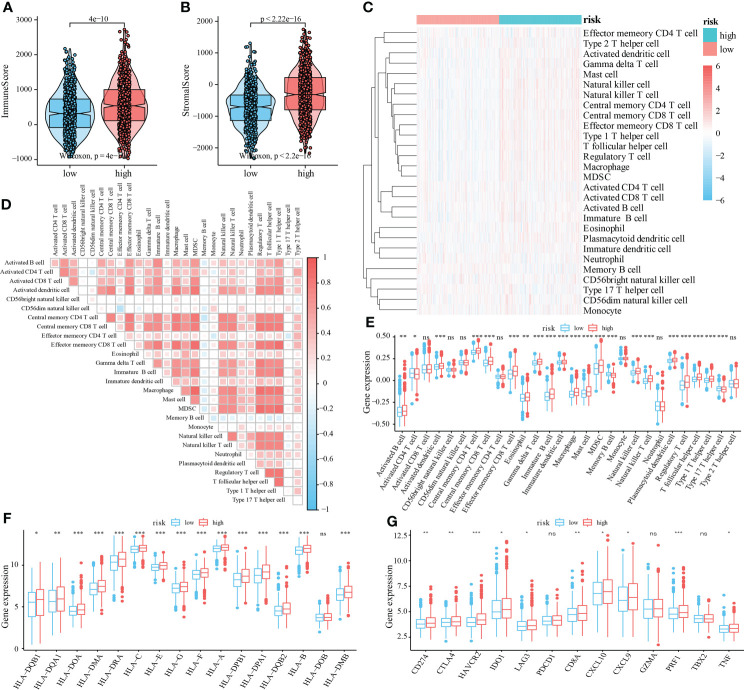
Relationship between mRNAsi-related metabolic risk score groups and infiltration of different immune cell subtypes. **(A, B)**. Differential analysis of immune scores and stromal scores between the high- and low-risk score group of patients with CRC. **(C)**. The heatmap showed the infiltration levels of 28 immune cell subtypes in CRC samples from TCGA and GEO datasets. **(D)**. Correlation heatmap showed the correlation between different levels of immune cell infiltration. **(E)**. Analysis of the difference of 28 levels of immune cell infiltration between two groups **(F)**. Multiple HLA family members, and **(G)** immunotherapy-related targets in high and low-risk score groups of patients with CRC. Differences were considered significant at *P < 0.05, **P < 0.01, ***P < 0.001, compared to the low-risk group. ns, not significant.

We then evaluated the immune contexture heterogeneity between the high and low-risk score groups. As shown in **Figure 6**, the immune and stromal scores of the high-risk score group were significantly higher than those in the low-risk score group (both *P <* 0.001, **Figures 6A, B**). In addition, to evaluate the degree of immune cell infiltration in tumor tissue, we used the ssGSEA algorithm and obtained the relative enrichment scores of 28 subtypes of immune cells between the two groups, as shown in the heatmap in **Figure 6C**. The correlation analysis showed that the infiltration levels of most immune cells were positively correlated (**Figure 6D**). Further analysis revealed that infiltration of CD4^+^T cells, CD8^+^ T cells, B cells, dendritic cells, eosinophils, mast cells, macrophages, myeloid-derived suppressor cells (MDSCs), natural killer cells, regulatory T cells, and T helper cells was higher in the high-risk score group (**Figure 6E**). In addition, in this study the expression of HLA family members and several immunotherapy-related target genes, such as *CD274 (PD-L1)*, *CTLA-4*, and *LAG-3*, was elevated in the tumor environment of the high-risk group compared with the low-risk group (**Figures 6F, G**).

### Sensitivity to chemotherapy and immunotherapy in high-risk and low-risk patients with CRC

**Figure 7:**
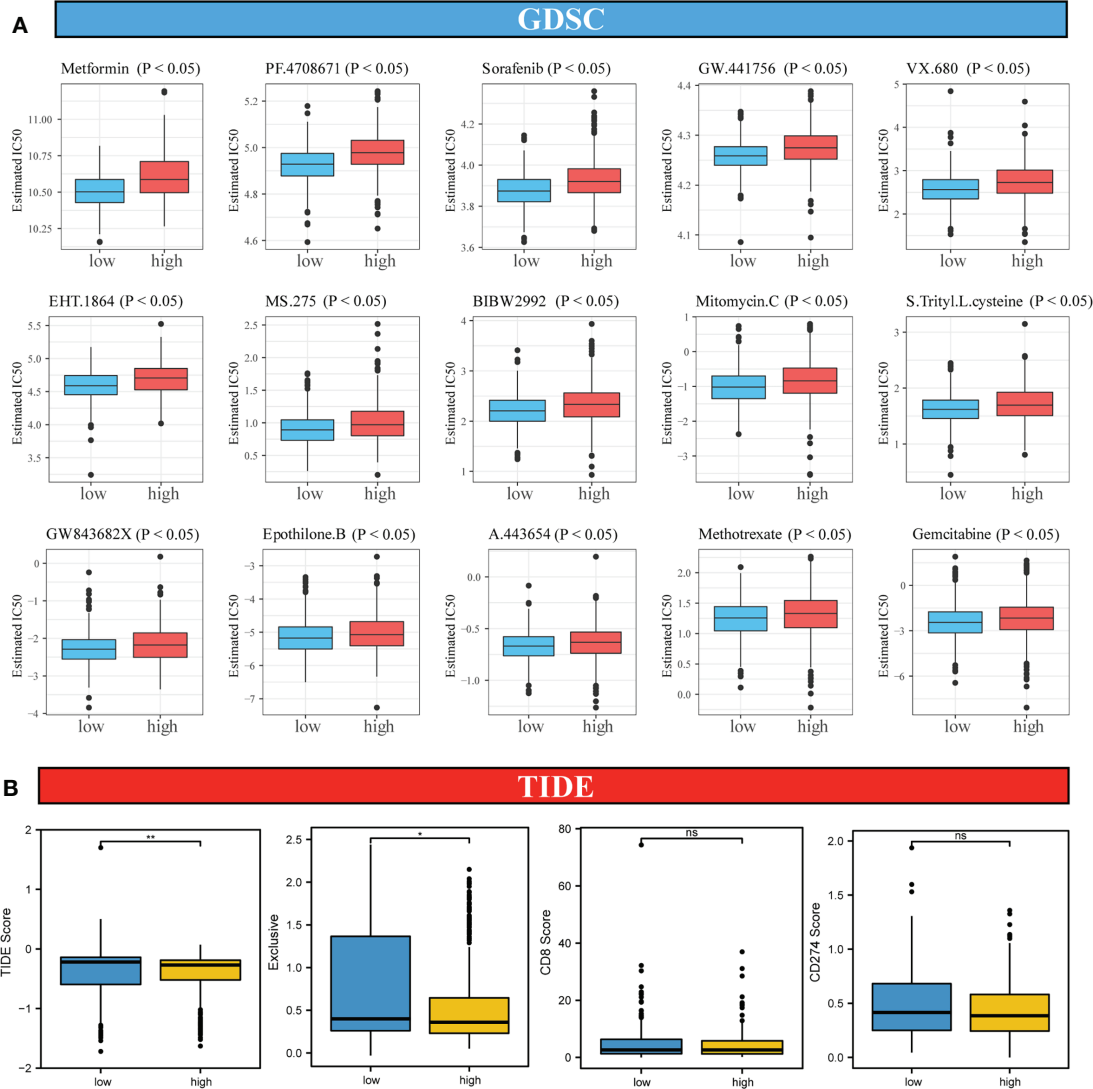
Analysis of sensitivity differences between high-risk and low-risk patients to different chemotherapeutic agents, small-molecule anticancer agents, and immunotherapy. **(A)**. Difference in sensitivity between high-risk and low-risk patients to 138 small-molecule anticancer agents and chemotherapeutic agents. **(B)**. Differences in TIDE score, immune exclusive, scores of immunotherapy targets, CD8 and CD274 in high-risk and low-risk score groups. Differences were considered significant at *P < 0.05, **P < 0.01, compared to the low-risk group. ns, not significant.

To analyze the differences in the sensitivity of patients with CRC to different drugs and small-molecule drugs based on the risk score, we downloaded the CRC cell line gene mutation data and the half-maximal inhibitory concentration (IC50) values of several anticancer drugs from the GDSC database. In GDSC, IC50 values for patients with CRC were predicted based on the responses of cell lines to 138 chemotherapeutic agents and small-molecule anticancer agents. This suggested that patients in the high-risk score group were less susceptible to multiple chemotherapeutic and small-molecule anticancer drugs, including Metformin, PF.4708671, Sorafenib, Mitomycin, Methotrexate, and gemcitabine (**Figure 7A**, all *P <* 0.05).

Because of the important role of immune-checkpoint inhibitor (ICI) therapy in tumors, we examined the sensitivity of two groups of patients with CRC to ICI therapy using the TIDE algorithm, which models two mechanisms of immune-evasion: T-cell dysfunction and reduced T-cell infiltration, to predict the immunotherapy response. As shown in **Figure 7B**, although no significant differences in the scores for two immune markers CD8 and CD274 between the high-risk and low-risk score groups was observed, the TIDE score in the high-risk score group was lower than that in the low-risk score group, suggesting a better response to the ICI therapy in the high-risk score group than in the low-risk score group.

### Analysis of genomic variation between high and low-risk score patients

Research has suggested that genomic variation affects tumor response to immunotherapy (38). Furthermore, we evaluated the differences in genomic variation in patients with CRC in the high and low-risk groups, including single nucleotide polymorphism (SNP), TMB, microsatellite instability (MSI), and CNV.

**Figure 8:**
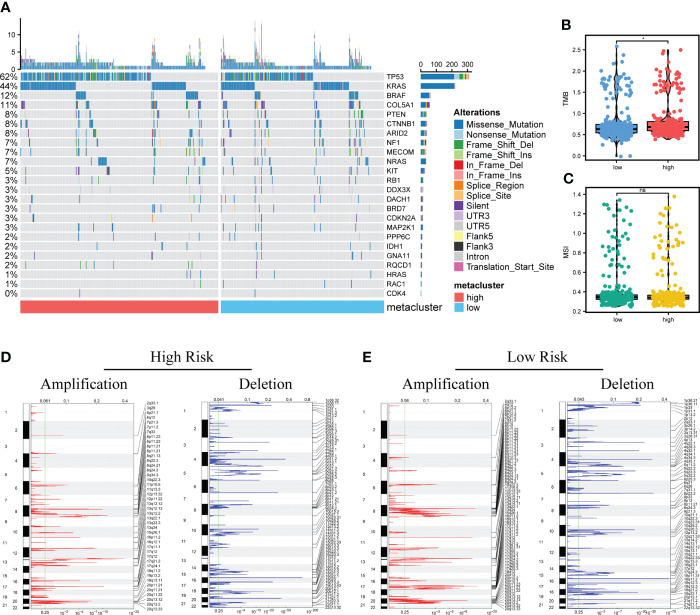
Analysis of genomic variation between high-risk and low-risk patients. **(A)**. Mutation profiles of common tumor-related genes in patients in high and low-risk score groups (Left: high-risk score group, right: low-risk score group). **(B, C)**. The difference of microsatellite instability (MSI) and tumor mutation burden (TMB) between two groups. **(D, E)**. Copy number variation in patients between two groups. Red indicates the amplified genes, and blue indicates the deleted genes. Differences were considered significant at *P < 0.05, compared with low-risk group. ns, not significant.

Difference in the level of the top SNP between the two groups was detected (**Figure 8A**). Furthermore, the TMB in the high-risk score group was higher than in the low-risk score group; however, no significant differences in MSI between the two groups were detected (**Figures 8B, C**). In addition, compared with the high-risk score group, the low-risk score group showed a significant increase in CNV, mainly characterized by deletion events (**Figures 8D, E**).

### Construction and validation of clinical prediction nomogram based on mRNAsi-related metabolic risk score model

**Figure 9:**
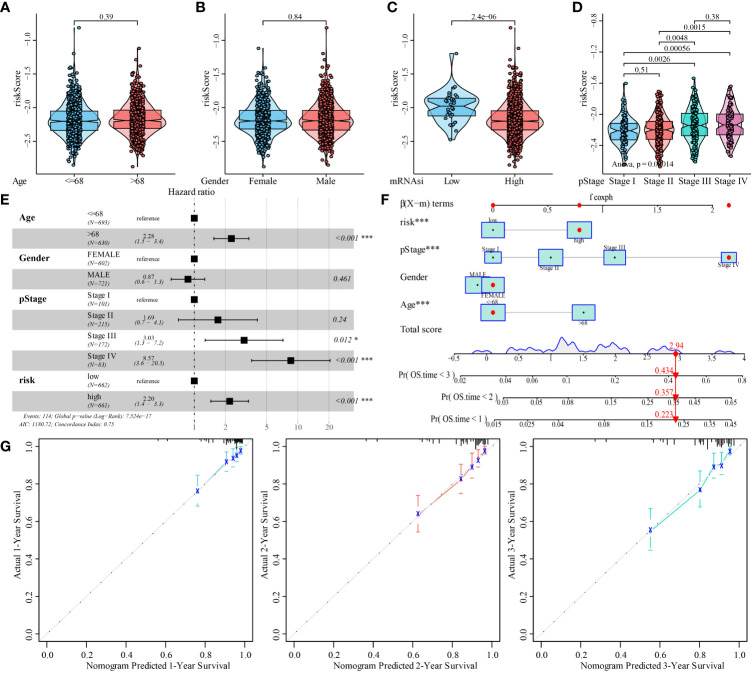
Analysis of the predictive ability of mRNAsi-related metabolic risk score model for the prognosis of patients with CRC. **(A–D)**. Analysis of correlation between mRNAsi-related metabolic risk scores and clinicopathological features of patients with CRC. **(E)**. Multivariate Cox regression analysis of HR and P values of risk score, combined with clinicopathological features. **(F)**. mRNAsi-related metabolic risk score combined with clinicopathological features to construct a clinical predictive model. **(G)**. The calibration curve of the nomogram showed that the risk score model had a good predictive ability for the overall survival rate of 1-, 2- and 3-year OS in patients. Differences were considered significant at *P < 0.05, ***P < 0.001, compared to the reference.

Next, we evaluated the association between mRNAsi-associated metabolic risk scores and clinicopathological characteristics in patients with CRC. The results showed no significant correlation between the risk scores and the age and gender of the patients (**Figures 9A, B**). However, high-risk scores were associated with lower mRNAsi and an advanced TNM state (**Figures 9C, D**).

In addition, univariate and multivariate Cox analyses showed that a high mRNAsi-related metabolic risk score was an independent predictor of prognosis in patients with CRC (**Figure 9E** and **Supplementary Table 5**). The mRNAsi-related metabolic risk score was then combined with different clinicopathological features to create a nomogram to predict OS in patients with CRC (**Figure 9F**). Then we preformed C-index to evaluate the differentiation of nomogram and found it has high discriminative ability (mean: 0.751 [range: 0.700–0.802]). In addition, the calibration curve showed that the 1-, 2-, and 3-year OS estimated by the nomogram matched with the actual OS values of the patients (**Figure 9G**).

### High-risk score group correlated with malignant progression, worse prognosis, inferior adjuvant chemotherapy responsiveness of CRC

To further determine the clinical significance of the risk score model in CRC, we evaluated the correlation between the high and low-risk score groups and the clinicopathological characteristics of patients with CRC in the Zhongshan Hospital cohort. The mRNA expression of 21 mRNAsi-related metabolic genes was measured by qRT-PCR. The median expression level of risk score was used as the cutoff value. Patients were divided into high and low-risk score groups. The high-risk score group was positively correlated with right-sided colon, poorer differentiation, ucoid adenocarcinoma and signet-ring cell carcinoma, nerve invasion, surgical margin positivity, and higher TNM stage (all *P <* 0.001, **Supplementary Table 6**). These findings suggest the gene set defining the high-risk score group is potentially involved in tumor progression.

**Figure 10:**
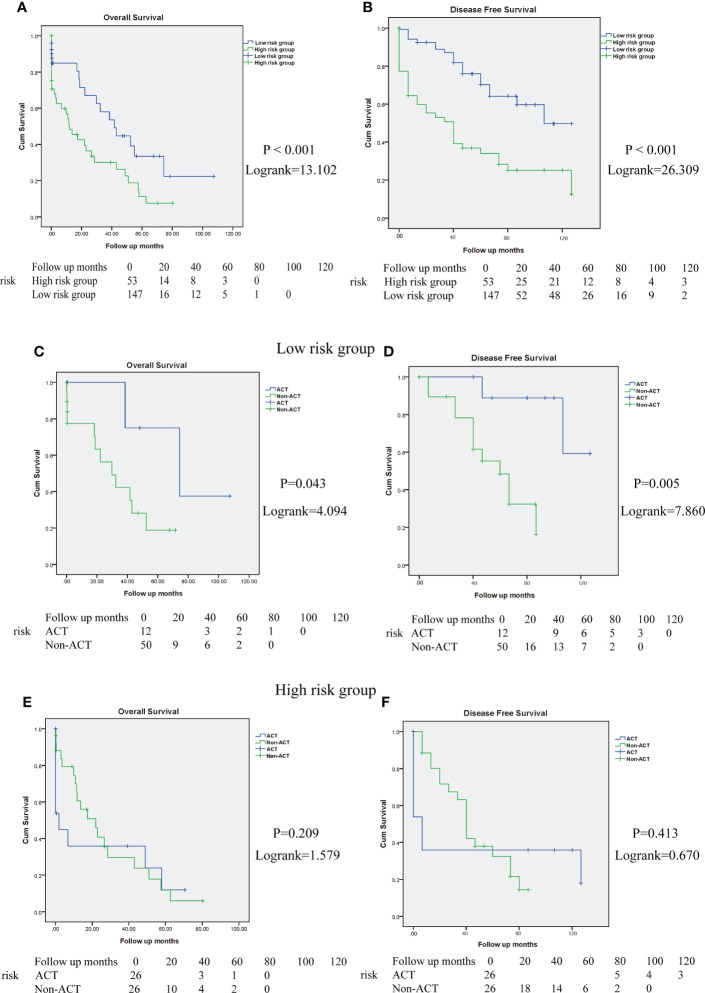
High-risk score group determines poor prognosis and impairs the ACT responsiveness of patients with CRC in Zhongshan cohort. **(A, B)**. Overall survival (OS) and disease-free survival (DFS) curves between high and low-risk score group in Zhongshan Hospital cohort. **(C, D)**. The OS and DFS curves for TNM stage III-IV patients with CRC in low-risk score group with or without ACT treatment. **(E, F)**. The OS and DFS curves for TNM stage III-IV patients with CRC in high-risk score group with or without ACT treatment.

To investigate the association between the risk score model and long-term outcomes of patients with CRC, Kaplan–Meier analysis was performed. The high-risk score group predicted worse survival of patients with CRC in the Zhongshan Hospital cohort (OS: *P <* 0.001, log-rank = 13.102; DFS: *P <* 0.001, log-rank = 26.309; **Figures 10A, B** **)**. These results indicate that the high-risk score was related to a worse outcome for patients with CRC.

In addition, we evaluated the interaction between the risk score model and therapeutic responsiveness to adjuvant chemotherapy (ACT) for TNM stage III-IV patients with CRC. In this study, ACT could improve patient survival in the low-risk score group (OS: *P =* 0.043, log rank =4.094, **Figure 10C**; DFS: *P =* 0.005, log rank = 7.860, **Figure 10D**) but had no significant benefit in the high-risk score group (OS: *P =* 0.209, log rank = 1.579, **Figure 10E**; DFS: *P =* 0.413, log rank = 0.670, **Figure 10F**). Therefore, these results suggest that the high-risk score group might have impaired therapeutic responsiveness to ACT in TNM stage III-IV CRC.

### High-risk score group shaped immunoevasive contexture

**Figure 11:**
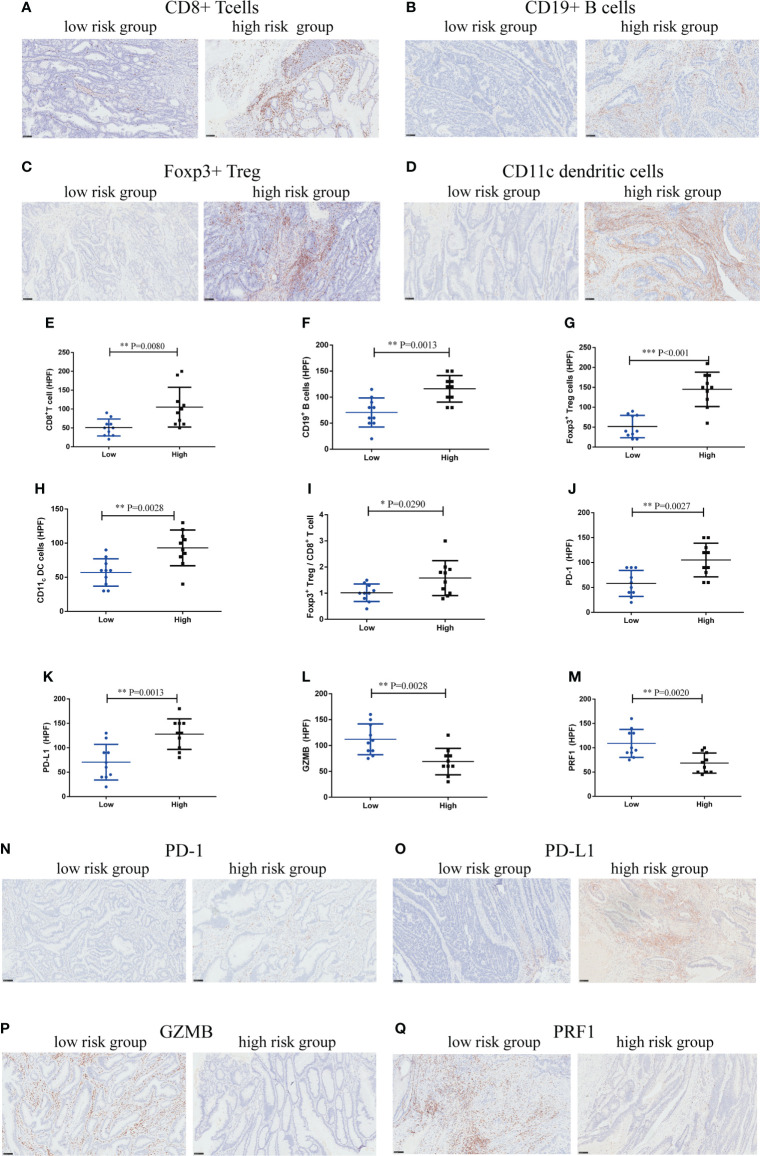
High-risk score group drives immunoevasive contexture and damages CD8^+^ T-cell function in CRC in Zhongshan cohort. **(A–D)**. Representative immunohistochemical (IHC) staining of four significant tumor-infiltrating immune cell subtypes between high and low-risk score groups, including CD8^+^ T cells, CD19^+^ B cells, Foxp3^+^ Tregs, CD11_c_ dendritic cells. **(E–I)**. Comparison of CD8^+^ T cells, CD19^+^ B cells, Foxp3^+^ Tregs, CD11_c_ dendritic cells and the ratio of Foxp3^+^ Tregs to CD8^+^ T cells between two groups. **(J–M)**. Expression of immunosuppressive checkpoints (PD-1, PD-L1) and effector molecules (GZMB, PRF1) between two groups. **(N–Q)**. Representative IHC staining of immunosuppressive checkpoints (PD-1, PD-L1) and effector molecules (GZMB, PRF1) between two groups. n = 10 in each group, scale bar: 250um. Differences were considered significant at *P < 0.05, **P < 0.01, ***P < 0.001, compared to the low-risk group.

To explore the underlying mechanism, we performed IHC staining of tumor-infiltrating immune cells in CRC tissues obtained from the Zhongshan Hospital cohort. The number of CD8^+^T cells (P=0.0080), CD19^+^B cells (P=0.0013), Foxp3^+^Tregs (P<0.001), and CD11c dendritic cells (P=0.0028) was more abundant in the high-risk group (**Figures 11A–H**). But the ratio of Foxp3^+^Treg cells to CD8^+^T cells also increased markedly in the high-risk score group (P=0.029) (**Figure 11I**), suggesting a more immunosuppressive tumor microenvironment with increased Treg cell infiltration. We further investigated whether the high-risk score group could affect CD8^+^T-cell function. The results indicated that CD8^+^T cells in the high-risk score group showed an exhausted T-cell phenotype with increased expression of immunosuppressive checkpoints, programmed cell death protein 1 (PD-1)(P=0.0027) and programmed cell death-ligand 1 (PD-L1)(P=0.0013), and decreased expression of CD8^+^T-cell effector molecules, granzyme B (GZMB)(P=0.0028) and perforin (PRF1)(P=0.0020), compared to the low-risk score group (**Figures 11J-Q**). Taken together, these data suggest that the high-risk score group may orchestrate an immunoevasive contexture and direct CD8^+^T-cell dysfunction in CRC.

## Discussion

Tumor recurrence and drug resistance have always been obstacles to the treatment of CRC. Studies have shown that CSCs and metabolic reprogramming promote immunosuppression, immune escape, and therapeutic resistance by interacting with immune cells (39, 40). Therefore, by integrating differential expression analysis between high and low mRNAsi, metabolism-related genes, co-expression network analysis, and LASSO Cox regression analysis, 21 mRNAsi-related metabolic genes with the highest prognostic value were identified and used to construct the risk score model. After validation first in the data retrieved from the TCGA and GEO databases, and then in the Zhongshan Hospital cohort, the CRC samples in the high-risk score group exhibited poor clinical outcome, increased immune-evasion, reduced sensitivity to chemotherapy, whereas potentially better response to immunotherapy, and higher genomic variation. This risk score model could be a tool to screen for patients with worse prognosis and inferior chemotherapy response, optimizing targeted treatment for CRC patients.

According to the GO functional enrichment results, most of these genes were clustered in functional groups related to the extracellular matrix (ECM). The ECM, as the main component of the tumor microenvironment, is considered to play a leading role in the progression of various cancers, including CRC, and promotes the invasion and metastasis of cancer (41–43). In addition, Ortensi et al. have demonstrated that the stemness characteristic of cancer tissue contributes to glioma invasiveness, which is closely related to the ECM (44). KEGG analysis also showed these genes were significantly abundant in inflammation, immunity, adhesion, invasion, and other processes. These results suggest that mRNAsi-related metabolic genes are involved in CRC metastasis and invasion.

Furthermore, GSEA revealed that hallmark hypoxia was significantly enriched in the high-risk score group. Our study and other studies have shown that hypoxia may promote glycolysis in CRC cells by activating the HIF-1α signaling pathway, thus promoting the proliferation and metastasis of CRC cells (45, 46). Hypoxia reduced the sensitivity of CRC to 5-fluorouracil chemotherapy (47). This suggests that mRNAsi-related metabolic genes participate in hypoxia-related pathways, leading to a poor prognosis and chemotherapy resistance in CRC.

Hub genes were obtained by PPI network construction and Cytoscape software analysis. GO semantic similarity analysis showed a key role for the *CALD1* gene. Li et al. found that CALD1 upregulated the expression of PD-L1 through the JAK/STAT signaling pathway and promoted malignant progression of bladder cancer (48). Several bioinformatics analyses and cellular studies have shown that CALD promoted the proliferation, metastasis, and invasion of CRC cells and is related to a reduction in OS (49). However, the exact mechanism of CALD involvement in CRC remains to be clarified.

Considering that the risk score model was derived from the stemness index and metabolism-related genes, which were significantly associated with antitumor immunity (13, 50), we further investigated the immune contexture heterogeneity between the high-risk and low-risk score groups. Tumor-infiltrating immune cell analysis showed that the high-risk score group had greater infiltration of CD8+ T cells, CD4+ T cells, B cells, Treg cells, dendritic cells, macrophages, MDSCs, neutrophils, regulatory T cells, and T helper cells, which is consistent with infiltrating immune cells from colorectal cancer in a highly inflammatory state (51). Meanwhile, Zhongshan cohort validated that CD8^+^T cells, CD19^+^B cells, Foxp3^+^Tregs, and CD11c dendritic cells was more abundant in the high-risk group. But an increase in the ratio of Foxp3^+^Treg cells to CD8^+^T cells in high-risk group, which leads to an immunosuppressive microenvironment. Treg cells promote tumorigenesis and development by inhibiting adaptive anti-tumor immunity, which is the key mechanism of tumor immune escape (52). The ratio of Foxp3+Treg cells to CD8+T cells as is a better variable because they are more representative of the biological characteristics of infiltrating immune cells (53). The immune microenvironment of tumor is closely related to clinical outcome and drug resistance (54). Further immunostaining experiments confirmed CD8^+^T-cell dysfunction with decreased levels of cytotoxic molecules (GZMB and PRF1) and increased the expression of immune checkpoints (PD-1 and PD L1) in the high-risk score group, resulting in a highly exhausted state and impaired immune function. The effect of tumor on immune cells can lead to T cell anergy or dysfunction, which promotes tumor escape and therapeutic drug resistance (55). Our results suggested that high-risk group induced an immunoevasive contexture and impaired antitumor immunity, which explains the poor clinical prognosis.

Immunotherapy is a novel cancer treatment approach. Although the effect is significant, only a fraction of the patients responds to the treatment (56, 57). In the TCGA and GEO databases, patients with CRC with high-risk scores had higher expression of immunotherapeutic molecules (such as PD-L1, CTLA4, HAVCR2 and LAG3), suggesting that CRC with high-risk scores may be more likely to be affected by the immune-checkpoint pathway, inhibit the antitumor immune response, and lead to deterioration of prognosis. Similarly, the TIDE algorithm predicted that patients with high-risk scores were more sensitive to ICI therapy. In the Zhongshan Hospital cohort, we confirmed that CD8^+^T cells in high-risk score patients showed an exhausted T-cell phenotype with increased expression of the immunosuppressive checkpoint, PD-1 and PD-L1, compared with low-risk score patients. These findings also suggest that CRC patients with high-risk scores may clinically benefit from immunotherapy. The TMB score of patients with the high-risk score group is higher, suggesting that PD-1 blocking therapy has a certain curative effect on these patients (13). Low TMB is an important reason for patient resistance to immunotherapy (58). This provides a new approach for stratifying this subgroup of patients with CRC to identify those who may achieve a superior response to immunotherapy.

Because of the close relationship of the stemness index and chemotherapeutic drug resistance in cancer, we analyzed the predictive ability of the risk score to chemotherapeutic drug sensitivity and found that patients with high-risk scores were less susceptible to a variety of small molecular anticancer drugs and chemotherapeutic medicines in TCGA and GEO databases. In the Zhongshan Hospital cohort, our findings suggest that the high-risk score group might have impaired therapeutic responsiveness to ACT in TNM stage III-IV CRC. Some studies have shown that in CRC, tumor stem cells lead to chemotherapy resistance by inhibiting antiapoptotic gene expression and reducing mitochondrial transcription initiation (59, 60). Due to the heterogeneity of CRC, the response of patients to chemotherapy is different, even at the same stage (61). This further suggests that patients with high-risk scores are potentially more suitable for immunotherapy than for traditional chemotherapy. However, the exact correlation between the risk score and the response to anticancer treatment needs to be further explored in a larger CRC cohort.

Finally, to improve clinical application, mRNAsi-related metabolic risk scores were combined with different clinicopathological characteristics to construct a prognostic nomogram and verify the predictive ability of the nomogram in TCGA and GEO datasets. In the Zhongshan Hospital cohort, a high-risk score was related to malignant progression and worse clinical outcomes in patients with CRC. This risk score model contains 21 important prognostic genes and has never been reported to identify the immunoevasive subgroup of patients with CRC in previous publications related to the CRC stemness index (62). Furthermore, this risk score model could help molecular typing and screening of differential subgroups to optimize personalized treatment and facilitate clinical translation.

Our study bears limitations. First, the mechanism of crosstalk between mRNAsi and metabolic reprogramming remains unclear, and further experimental studies are needed. Second, the clinical data of patients receiving immunotherapy in this study were limited, and the robust ability of the risk score model to predict immunotherapy responsiveness needs to be verified in a future larger immunotherapy cohort. Finally, although the new model and nomogram could accurately forecast the survival of patients with CRC in the TCGA and GEO databases and the Zhongshan Hospital cohort, more cell experiments, animal models, and clinical samples are needed to verify the value of this mRNAsi-related metabolic risk score model before developing immunotherapy strategies for subgroups of patients with CRC.

In this study, we proposed and validated a new risk score model according to 21 mRNAsi-related metabolic genes. The high-risk score group had a poorer clinical prognosis, inferior sensitivity to chemotherapy, a potentially better response to immunotherapy, and an immunoevasive environment, which sheds light on more accurate risk stratification and divides subgroups of patients with CRC for immunotherapy.

## Data availability statement

The datasets presented in this study can be found in online repositories. The names of the repository/repositories and accession number(s) can be found in the article/**Supplementary Material**.

## Ethics statement

The studies involving human participants were reviewed and approved by This study was approved by the ethics committee of Zhongshan Hospital, Fudan University (B2022-068R2). The patients/participants provided their written informed consent to participate in this study.

## Author contributions

MLW and CHM designed the study. MLW, TL, JZ, MMG, and WLZ, performed the study. MLW, WCG, CHS, QWL, YY, KN, and ZWZ analyzed the data. MLW and TL wrote the paper MLW, MLS and DZ revised the paper. All authors contributed to the article and approved the submitted version.

## Funding

Our study was supported by the National Natural Science Foundation of China (No.82002538, 82072213); Shanghai Pujiang Talent Plan (No. 2020PJD013); Clinical Research Plan of SHDC (No. SHDC2020CR1005A); and National Key Research and Development Program of China (No. 2020YFC2008400).

## Acknowledgments

We thank Rongkui Luo (Department of Pathology, Zhongshan hospital Fudan University) for providing technical consultation.

## Conflict of interest

The authors declare that the research was conducted in the absence of any commercial or financial relationships that could be construed as a potential conflict of interest.

## Publisher’s note

All claims expressed in this article are solely those of the authors and do not necessarily represent those of their affiliated organizations, or those of the publisher, the editors and the reviewers. Any product that may be evaluated in this article, or claim that may be made by its manufacturer, is not guaranteed or endorsed by the publisher.

## Supplementary material

Supplementary Figure 1 Data collection and processing. Box plot of CRC samples in the GSE17536 **(A)** and GSE39582 **(B)** datasets. Box plots of TCGA, GSE17536, and GSE39582 datasets before **(C)** and after **(D)** merging and de-batching.

Supplementary Figure 2 GSEA of DEGs in high and low-risk score groups of patients with CRC. **(A–I)**. GSEA analysis of DEGs in high and low-risk score groups of patients with CRC.

Supplementary Figure 3
**(A)**. The heatmap showing the expression of metabolism-related DEGs in patients with CRC. **(B)**. The heatmap shows the expression of 83 significantly differentially expressed mRNAsi-related metabolic genes in the CRC and normal tissues.

Supplementary Table 1 Association between risk score and clinical characteristics of patients with CRC.

Supplementary Table 2 GO analysis of DEGs in high and low-risk score groups of patients with CRC.

Supplementary Table 3 KEGG analysis of DEGs in high and low-risk score groups of patients with CRC.

Supplementary Table 4 GSEA of DEGs in high and low-risk score groups of patients with CRC.

Supplementary Table 5 Univariate and multivariate Cox analyses of OS prediction in patients with CRC based on mRNAsi-related metabolic risk score model.

Supplementary Table 6 Relationship between high and low-risk score groups and clinicopathological features of patients with CRC in Zhongshan Hospital cohort.

Supplementary Table 7 Primers used in reverse transcription PCR analysis.

Supplementary Table 8 Immunohistochemistry antibodies.
