# Supplementary figures and images for: mRNAsi-related metabolic risk score model identifies poor prognosis, immunoevasive contexture, and low chemotherapy response in colorectal cancer patients through machine learning
Source: Front Immunol. 2022 Aug 23;13:950782. doi: 10.3389/fimmu.2022.950782 (PMC9445443; doi:10.3389/fimmu.2022.950782)

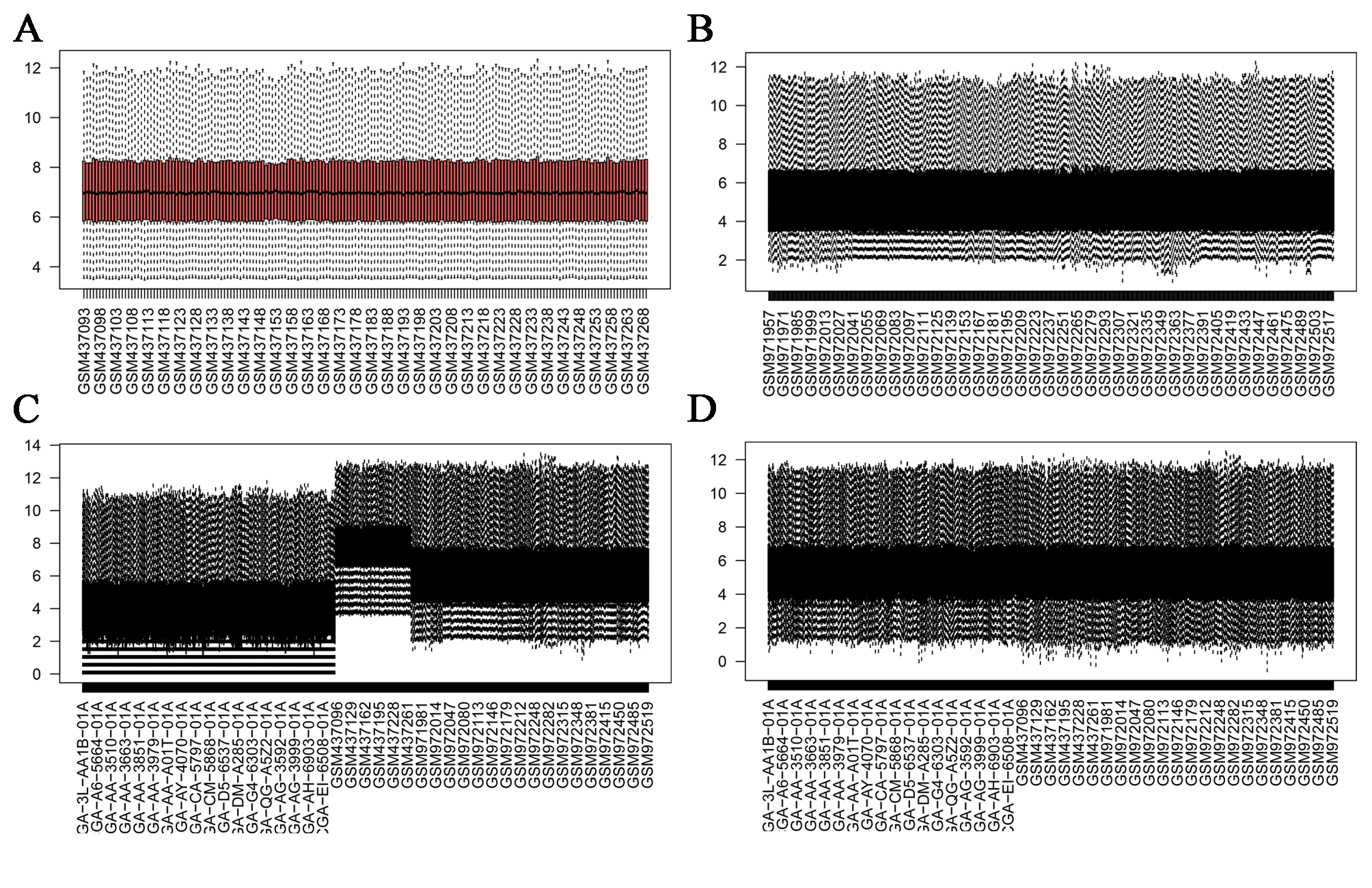

Supplement: Supplementary Figure 1 — Data collection and processing. Box plot of CRC samples in the GSE17536 (A) and GSE39582 (B) datasets. Box plots of TCGA, GSE17536, and GSE39582 datasets before (C) and after (D) merging and de-batching. [file Image_1.tif]

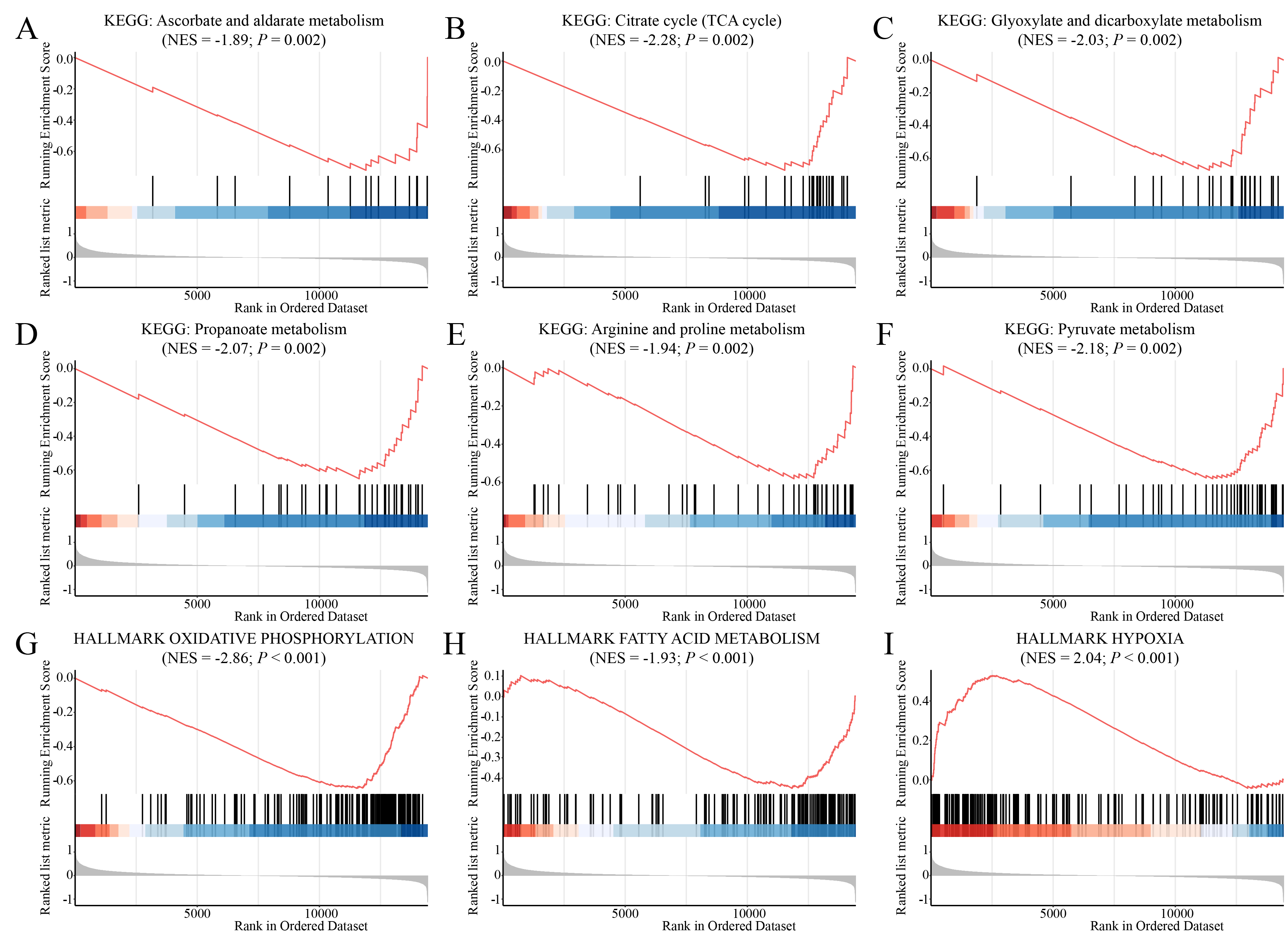

Supplement: Supplementary Figure 2 — GSEA of DEGs in high and low-risk score groups of patients with CRC. (A–I). GSEA analysis of DEGs in high and low-risk score groups of patients with CRC. [file Image_2.tif]

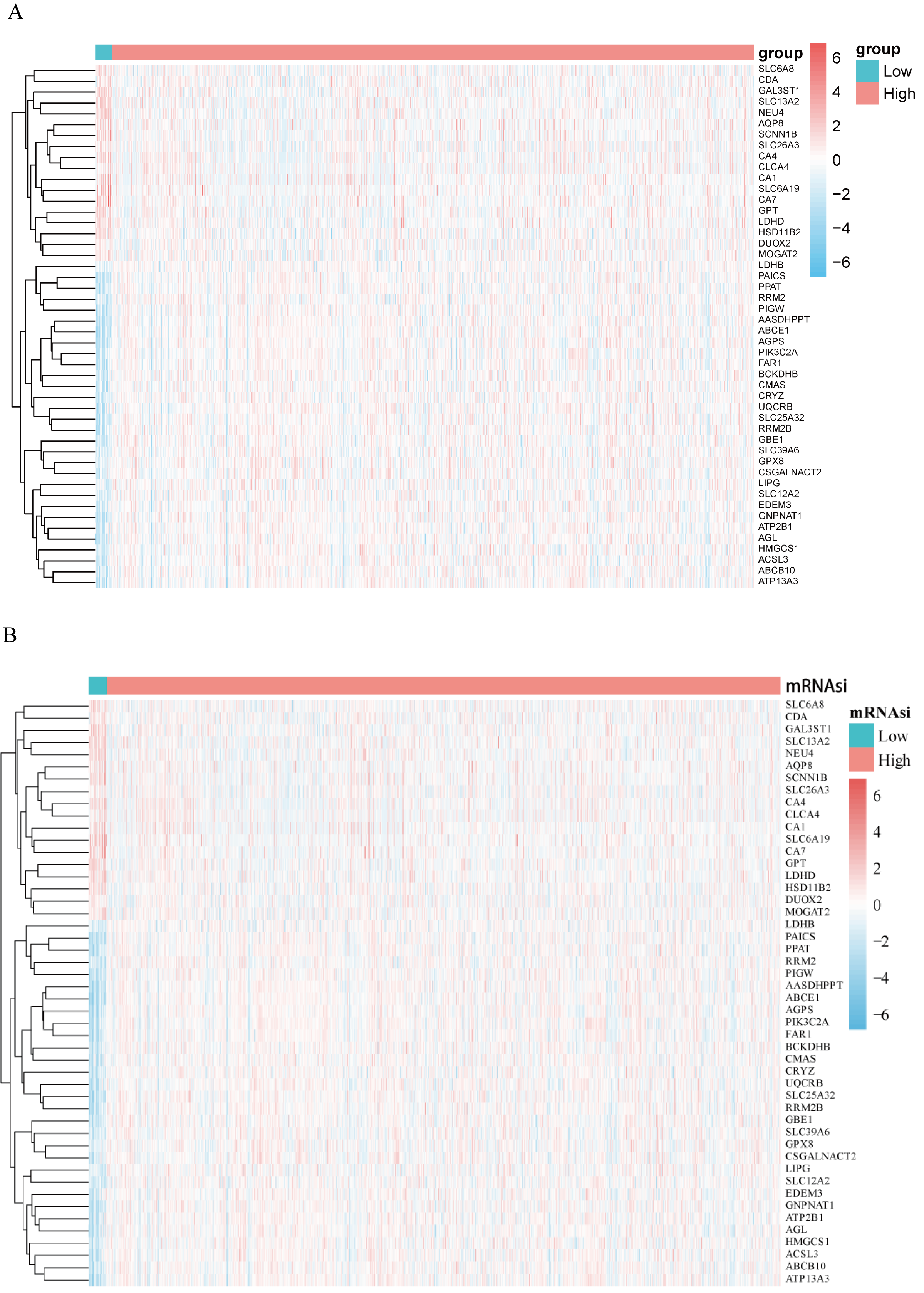

Supplement: Supplementary Figure 3 — (A). The heatmap showing the expression of metabolism-related DEGs in patients with CRC. (B). The heatmap shows the expression of 83 significantly differentially expressed mRNAsi-related metabolic genes in the CRC and normal tissues. [file Image_3.tif]
