# Supplementary material for: mRNAsi-related metabolic risk score model identifies poor prognosis, immunoevasive contexture, and low chemotherapy response in colorectal cancer patients through machine learning
Source: Front Immunol. 2022 Aug 23;13:950782. doi: 10.3389/fimmu.2022.950782 (PMC9445443; doi:10.3389/fimmu.2022.950782)
Supplement: Supplementary Table 1 — Association between risk score and clinical characteristics of patients with CRC. [file Table_1.docx]

| Variables | All patients  (n = 1323) | High risk  (n = 661) | Low risk  (n = 662) | *P* value |
| --- | --- | --- | --- | --- |
| Age |  |  |  | 0.528 |
| ＜68 | 693 (52.4%) | 340 (51.4%) | 353 (53.3%) |  |
| ≥68 | 630 (47.6%) | 321 (48.6%) | 309 (46.7%) |  |
| Gender |  |  |  | 0.888 |
| Female | 602 (45.5%) | 299 (45.2%) | 303 (45.8%) |  |
| Male | 721 (54.5%) | 362 (54.8%) | 359 (54.2%) |  |
| TNM Stage |  |  |  | 0.014* |
| Stage I | 101 (17.7%) | 46 (15.2%) | 55 (20.5%) |  |
| Stage II | 215 (37.7%) | 103 (34.0%) | 112 (41.8%) |  |
| Stage III | 172 (30.1%) | 101 (33.3%) | 71 (26.5%) |  |
| Stage IV | 83 (14.5%) | 53 (17.5%) | 30 (11.2%) |  |

**Supplementary Table 1.** Association between risk score and clinical characteristics of patients with CRC

TNM stage: tumor node metastasis (TNM) stage; Differences were considered significant at *, P<0.05
