# Supplementary material for: mRNAsi-related metabolic risk score model identifies poor prognosis, immunoevasive contexture, and low chemotherapy response in colorectal cancer patients through machine learning
Source: Front Immunol. 2022 Aug 23;13:950782. doi: 10.3389/fimmu.2022.950782 (PMC9445443; doi:10.3389/fimmu.2022.950782)
Supplement: Supplementary Table 2 — GO analysis of DEGs in high and low-risk score groups of patients with CRC. [file Table_2.docx]

| Category | Pathway ID | Pathway description | Count in gene set | P value |
| --- | --- | --- | --- | --- |
| GOTERM_BP | GO:0030198 | extracellular matrix organization | 45 | 1.14E-31 |
| GOTERM_BP | GO:0043062 | extracellular structure organization | 45 | 1.28E-31 |
| GOTERM_BP | GO:0030199 | collagen fibril organization | 16 | 2.67E-18 |
| GOTERM_CC | GO:0062023 | collagen-containing extracellular matrix | 58 | 3.22E-45 |
| GOTERM_CC | GO:0005788 | endoplasmic reticulum lumen | 28 | 1.38E-16 |
| GOTERM_CC | GO:0005581 | collagen trimer | 17 | 3.98E-16 |
| GOTERM_MF | GO:0005201 | extracellular matrix structural constituent | 37 | 7.58E-36 |
| GOTERM_MF | GO:0005539 | glycosaminoglycan binding | 27 | 1.77E-18 |
| GOTERM_MF | GO:0030020 | extracellular matrix structural constituent conferring tensile strength | 10 | 7.83E-11 |

**Supplementary Table 2.** GO analysis of differentially expressed genes in high and low risk score groups of patients with CRC

BP: biological processes; MF: molecular functions; CC: cellular components.
