# Supplementary material for: mRNAsi-related metabolic risk score model identifies poor prognosis, immunoevasive contexture, and low chemotherapy response in colorectal cancer patients through machine learning
Source: Front Immunol. 2022 Aug 23;13:950782. doi: 10.3389/fimmu.2022.950782 (PMC9445443; doi:10.3389/fimmu.2022.950782)
Supplement: Supplementary Table 3 — KEGG analysis of DEGs in high and low-risk score groups of patients with CRC. [file Table_3.docx]

| **Category** | **Pathway ID** | **Pathway description** | **Count in gene set** | **P value** |
| --- | --- | --- | --- | --- |
| KEGG_Pathway | hsa04974 | Protein digestion and absorption | 12 | 2.82E-08 |
| KEGG_Pathway | hsa04145 | Phagosome | 13 | 2.96E-07 |
| KEGG_Pathway | hsa04610 | Complement and coagulation cascades | 10 | 3.91E-07 |
| KEGG_Pathway | hsa04512 | ECM-receptor interaction | 10 | 5.43E-07 |
| KEGG_Pathway | hsa04510 | Focal adhesion | 14 | 1.25E-06 |
| KEGG_Pathway | hsa05150 | Staphylococcus aureus infection | 8 | 7.65E-05 |
| KEGG_Pathway | hsa04933 | AGE-RAGE signaling pathway in diabetic complications | 7 | 0.00063526 |
| KEGG_Pathway | hsa05205 | Proteoglycans in cancer | 10 | 0.0008257 |
| KEGG_Pathway | hsa05133 | Pertussis | 5 | 0.00503315 |

**Supplementary Table 3**. KEGG analysis of differentially expressed genes in high and low risk score groups of patients with CRC

KEGG: Kyoto Encyclopedia of Genes and Genomes.
