# Supplementary material for: mRNAsi-related metabolic risk score model identifies poor prognosis, immunoevasive contexture, and low chemotherapy response in colorectal cancer patients through machine learning
Source: Front Immunol. 2022 Aug 23;13:950782. doi: 10.3389/fimmu.2022.950782 (PMC9445443; doi:10.3389/fimmu.2022.950782)
Supplement: Supplementary Table 4 — GSEA of DEGs in high and low-risk score groups of patients with CRC. [file Table_4.docx]

**Supplementary Table 4**. GSEA of differentially expressed genes in high and low risk score groups of patients with CRC.

| Name | Size | Enrichment  Score | NES | P value | Leading edge |
| --- | --- | --- | --- | --- | --- |
| Ascorbate and aldarate metabolism | 15 | -0.7209832 | -1.8866919 | 0.00253807 | tags=60%, list=18%, signal=50% |
| Citrate cycle (TCA cycle) | 28 | -0.736128 | -2.2757112 | 0.00257732 | tags=79%, list=20%, signal=63% |
| Glyoxylate and dicarboxylate metabolism | 26 | -0.6722132 | -2.0291356 | 0.00263158 | tags=73%, list=21%, signal=58% |
| Propanoate metabolism | 31 | -0.6517447 | -2.0650798 | 0.00263158 | tags=58%, list=19%, signal=47% |
| Arginine and proline metabolism | 40 | -0.5802105 | -1.9363978 | 0.00294985 | tags=50%, list=18%, signal=41% |
| Pyruvate metabolism | 42 | -0.6501703 | -2.1779657 | 0.00294985 | tags=64%, list=20%, signal=51% |
| HALLMARK_OXIDATIVEPHOSPHORYLATION | 178 | -0.6442579 | -2.8564541 | 1.00E-10 | tags=63%, list=20%, signal=51% |
| HALLMARK_FATTY_ACID_METABOLISM | 141 | -0.4503071 | -1.9292611 | 8.41E-07 | tags=47%, list=18%, signal=39% |
| HALLMARK_HYPOXIA | 191 | 0.52892489 | 2.04112895 | 1.90E-09 | tags=46%, list=19%, signal=38% |
