# Supplementary material for: mRNAsi-related metabolic risk score model identifies poor prognosis, immunoevasive contexture, and low chemotherapy response in colorectal cancer patients through machine learning
Source: Front Immunol. 2022 Aug 23;13:950782. doi: 10.3389/fimmu.2022.950782 (PMC9445443; doi:10.3389/fimmu.2022.950782)
Supplement: Supplementary Table 5 — Univariate and multivariate Cox analyses of OS prediction in patients with CRC based on mRNAsi-related metabolic risk score model. [file Table_5.docx]

|  | **Univariate Cox analysis** | | | | | **Multivariate Cox analysis** | | | | | | |
| --- | --- | --- | --- | --- | --- | --- | --- | --- | --- | --- | --- | --- |
|  | **HR** | **HR.95L** | | **HR.95H** | ***P* value** | **HR** | | **HR.95L** | | **HR.95H** | ***P* value** | |
| Age (>68 vs. ≤68) | 1.63 | | 1.33 | 1.99 | 2.76E-06 | | 2.28 | | 1.55 | 3.37 | | 3.25E-05 |
| Gender (Male vs. Female) | 1.18 | | 0.96 | 1.45 | 0.10611942 | | 0.87 | | 0.60 | 1.26 | | 0.46129337 |
| pStage (Stage II vs. Stage I) | 1.76 | | 0.73 | 4.23 | 0.20644641 | | 1.69 | | 0.70 | 4.07 | | 0.24038462 |
| pStage (Stage III vs. Stage I) | 3.45 | | 1.46 | 8.15 | 0.00482074 | | 3.03 | | 1.27 | 7.21 | | 0.01213793 |
| pStage (Stage IV vs. Stage I) | 8.88 | | 3.75 | 21.02 | 6.82E-07 | | 8.57 | | 3.58 | 20.50 | | 1.38E-06 |
| Risk score (High risk vs. Low risk) | 2.37 | | 1.92 | 2.94 | 1.34E-15 | | 2.20 | | 1.45 | 3.34 | | 0.00023066 |

**Supplementary Table 5**. Univariate and multivariate Cox analyses of overall survival prediction in patients with CRC based on mRNAsi-related metabolic risk score model

HR: hazard ratio
