# Supplementary material for: mRNAsi-related metabolic risk score model identifies poor prognosis, immunoevasive contexture, and low chemotherapy response in colorectal cancer patients through machine learning
Source: Front Immunol. 2022 Aug 23;13:950782. doi: 10.3389/fimmu.2022.950782 (PMC9445443; doi:10.3389/fimmu.2022.950782)
Supplement: Supplementary Table 6 — Relationship between high and low-risk score groups and clinicopathological features of patients with CRC in Zhongshan Hospital cohort. [file Table_6.docx]

**Supplementary Table 6**. Relationship between high and low risk score groups and clinicopathological features of patients with CRC in Zhongshan Hospital Cohort

| Characters | level | Low risk group | High risk group | P value |
| --- | --- | --- | --- | --- |
| n |  | 147 | 53 |  |
| Age, n(%) | <=44 | 14(9.5%) | 8(15.1%) | 0.074 |
|  | 45-54 | 31(21.1%) | 17(32.1%) |  |
|  | 55-64 | 52(35.4%) | 19(35.8%) |  |
|  | 65-74 | 31(21.1%) | 8(15.1%) |  |
|  | >=75 | 19(12.9%) | 1(1.9%) |  |
| Gender, n(%) | Female | 66(44.9%) | 23(43.4%) | 0.850 |
|  | Male | 81(55.1%) | 30(56.6%) |  |
| Tumor location, n(%) | Rectum | 65(44.2%) | 20(37.7%) | <0.040 |
|  | Left-side colon | 45(30.6%) | 11(20.8%) |  |
|  | Right-side colon  Entire colon | 33(22.4%)  4(2.7%) | 22(41.5%)  0(0.0%) |  |
| Tumor differentiation, n(%) | Well | 8(5.4%) | 0(0.0%) | <0.001 |
|  | Moderate | 77(52.4%) | 31(58.5%) |  |
|  | Poor  Unknown | 17(11.6%)  45(30.6%) | 21(39.6%)  1(1.9%) |  |
| Tumor histology, n(%) | adenocarcinoma | 133(90.5%) | 38(71.7%) | 0.001 |
|  | ucoid adenocarcinoma | 14(9.5%) | 13(24.5%) |  |
|  | signet-ring cell carcinoma | 0(0.0%) | 2(3.8%) |  |
| Nerve invasion, n(%) | No | 136(92.5%) | 25(47.2%) | <0.001 |
|  | Yes | 11(7.5%) | 28(52.8%) |  |
| Surgical margin positive, n(%) | No | 147(100%) | 49(92.5%) | 0.001 |
|  | Yes | 0(0.0%) | 4(7.5%) |  |
| TNM stage, n(%) | Stage I | 49(33.3%) | 1(1.9%) | <0.001 |
|  | Stage II | 36(24.5%) | 0(0.0%) |  |
|  | Stage III | 41(27.9%) | 22(41.5%) |  |
|  | Stage IV  Unknown | 14(9.5%)  7(4.8%) | 30(56.6%)  0(0.0%) |  |

TNM stage: tumor node metastasis stage.
