# Supplementary material for: mRNAsi-related metabolic risk score model identifies poor prognosis, immunoevasive contexture, and low chemotherapy response in colorectal cancer patients through machine learning
Source: Front Immunol. 2022 Aug 23;13:950782. doi: 10.3389/fimmu.2022.950782 (PMC9445443; doi:10.3389/fimmu.2022.950782)
Supplement: Supplementary Table 8 — Immunohistochemistry antibodies. [file Table_8.docx]

**Supplementary Table 8. Immunohistochemistry antibodies**

| **Antibody name** | **Description** | **Manufacturer** | **Catalog No.** | **Dilution** | **Identical cells** |
| --- | --- | --- | --- | --- | --- |
| anti-CD8 alpha antibody | Rabbit monoclonal | Abcam | Ab237709 | 1:200 | CD8^+^ T cells |
| Anti-Foxp3 antibody | Mouse monoclonal | Abcam | Ab450 | 1:50 | Tregs (regulatory T cells) |
| Anti-CD19 antibody | Rabbit  monoclonal | Abcam | Ab99965 | 1:200 | CD19^+^ B cells |
| Anti-CD11c antibody | Mouse  monoclonal | Abcam | Ab218434 | 1:200 | DC cells |
| Anti-PD-1 antibody | Rabbit monoclonal | Abcam | Ab137132 | 1:250 | PD-1^+^ cells |
| Anti-PD-L1 antibody | Rabbit monoclonal | Abcam | Ab213524 | 1:250 | PD-L1^+^ cells |
| Anti-GZMB antibody | Rabbit monoclonal | Abcam | Ab243879 | 1:100 | GZMB^+^ cells |
| Anti-PRF1 antibody | Mouse monoclonal | Abcam | Ab75573 | 1:200 | PRF1^+^ cells |
