# Supplementary material for: mRNAsi-related metabolic risk score model identifies poor prognosis, immunoevasive contexture, and low chemotherapy response in colorectal cancer patients through machine learning
Source: Front Immunol. 2022 Aug 23;13:950782. doi: 10.3389/fimmu.2022.950782 (PMC9445443; doi:10.3389/fimmu.2022.950782)
Supplement: Supplementary file 12 [file Table_9.docx]

**Supplementary Table 9. Primers used in RT-PCR analysis**

| **Gene** | **Primer Sequence** |
| --- | --- |
| PAICS(human) | 5’-TTGCAGAAGAATAGCAACTGGTT-3’ |
|  | 5’-CACTGTGGGTCATTATTGGCAT-3’ |
| PTGES3(human) | 5’-TGTTTGCGAAAAGGAGAATCCG-3’ |
|  | 5’-CCATGTGATCCATCATCTCAGAG-3’ |
| GNPNAT1(human) | 5’-ACTCCTATGTTTGACCCAAGTCT-3’ |
|  | 5’-TCTGTTAGCTGACCCAATACCT-3’ |
| PGM3(human) | 5’-GCAGAGAGTGCTTATTGACATCA-3’ |
|  | 5’-TGTGAAAGTTTCTCACTGCTGG-3’ |
| MTHFD2(human) | 5’-CTGCGACTTCTCTAATGTCTGC-3’ |
|  | 5’-CTCGCCAACCAGGATCACA-3’ |
| MTAP(human) | 5’-ACCACCGCCGTGAAGATTG-3’ |
|  | 5’-GCATCAGATGGCTTGCCAA-3’ |
| DCK(human) | 5’-CCATCGAAGGGAACATCGCT-3’ |
|  | 5’-GGTAAAAGACCATCGTTCAGGT-3’ |
| GBE1(human) | 5’-GGAGATCGACCCGTACTTGAA-3’ |
|  | 5’-ACATCTGTGGACGCCAAATGA-3’ |
| SLC25A36(human) | 5’-GTGGTGGTACAGTGGGAGCTA-3’ |
|  | 5’-GGAGACACTACTCGGTTGACA-3’ |
| KCTD3(human) | 5’-GAGATCGTCCAACTGAACGTAG-3’ |
|  | 5’-TTCTGCTTCATGCCTGAGAAC-3’ |
| ACADSB(human) | 5’-TCTTGGGACAAATTGGACATGG-3’ |
|  | 5’-GTGAGCCACTTGGTGTTGGA-3’ |
| ABCD3(human) | 5’-TACTTGACGGCGCGAAACTC-3’ |
|  | 5’-CACCACAGCTCGCTCCTTTT-3’ |
| BCKDHB(human) | 5’-TGGAGTCTTTAGATGCACTGTTG-3’ |
|  | 5’-CGCAATTCCGATTCCAAATCCAA-3’ |
| FUT4(human) | 5’-GATCTGCGCGTGTTGGACTA-3’ |
|  | 5’-GAGGGCGACTCGAAGTTCAT-3’ |
| PHOSPHO2(human) | 5’-ATCATAGACGACAACAGTGACAC-3’ |
|  | 5’-GCTTTTACCCCCTCATCTCTCAA-3’ |
| EDEM3(human) | 5’-CGAGCCCATGAGTAGGGAG-3’ |
|  | 5’-AAAGGCATGAGTTCATCAGCA-3’ |
| SLC16A1(human) | 5’-AGGTCCAGTTGGATACACCCC-3’ |
|  | 5’-GCATAAGAGAAGCCGATGGAAAT-3’ |
| SLC6A8(human) | 5’-GGCCTGGGGCTTCTATTACC-3’ |
|  | 5’-CAGCCTCAAGACTTTGTTCTCC-3’ |
| ELOVL7(human) | 5’-GCCAAGTGACTTTCCTTCATGT-3’ |
|  | 5’-GGCGACAATAACAAACTGGACA-3’ |
| NEU4 | 5’-GGCCACGGGATGACAGTTG-3’ |
|  | 5’-CAGGCGGATACCCATGTGTAG-3’ |
| RRM2 | 5’-CACGGAGCCGAAAACTAAAGC-3’ |
|  | 5’-TCTGCCTTCTTATACATCTGCCA-3’ |
| GAPDH(human) | 5’-GGAGCGAGATCCCTCCAAAAT-3’ |
|  | 5’-GGCTGTTGTCATACTTCTCATGG-3’ |
